# A novel biliary-specific near-infrared fluorescent dye (BL-760) enhances visualization of the biliary tree in a swine inflammatory model of bile duct obstruction

**DOI:** 10.1007/s00464-025-12252-9

**Published:** 2025-10-06

**Authors:** Alex I. Halpern, Kwan M. Yang, Khalid M. Ali, Bo Ning, Martin J. Schnermann, Anthony D. Sandler, Richard J. Cha

**Affiliations:** 1 Sheikh Zayed Institute for Pediatric Surgical Innovation, Children’s National Hospital, 111 Michigan Avenue, Washington, DC 20010 USA; 2 Department of General and Thoracic Surgery, Children’s National Hospital, Washington, DC USA; 3 George Washington University School of Medicine and Health Sciences, Washington, DC USA; 4 Department of Surgery, Gangneung Asan Hospital, University of Ulsan College of Medicine, Gangneung, Republic of Korea; 5 Chemical Biology Laboratory, Center for Cancer Research, National Cancer Institute, Frederick, MD USA

**Keywords:** Cholecystectomy, Bile duct injury, Indocyanine green, Near-infrared dye, BL-760

## Abstract

**Background:**

Bile duct injury (BDI) is a morbid complication of laparoscopic cholecystectomy due to poor recognition of the anatomy and inadequate visualization of the extra-hepatic biliary ducts. Near-infrared indocyanine green (ICG) is the most commonly used non-invasive option to assist with identification of the extra-hepatic biliary structures. However ICG is limited by its slow onset of action and lack of specificity for the biliary tree. In light of these limitations our team previously reported bile-label 760 (BL-760), a pre-clinical near-infrared dye, as a novel tool for intraoperative identification of biliary structures. This study builds upon our previous work and assesses the intraoperative detection of the extra-hepatic biliary ducts in a swine model of biliary obstruction using intravenously administered BL-760.

**Methods:**

A survival swine study utilizing BL-760 was performed in two 30 kg female Yorkshire swine. Each swine underwent two surgeries. In the initial surgeries, laparoscopic clipping of an extra-hepatic biliary duct was performed under BL-760 guidance. The cystic duct (CD) was clipped in Swine #1 and the common bile duct (CBD) was clipped in Swine #2. On the third postoperative day, a laparoscopic cholecystectomy was performed in each swine under BL-760 guidance. Target-to-background ratios (TBRs) of the extra-hepatic biliary ducts to the liver were measured using ImageJ.

**Results:**

The surgeries were performed without complication. The TBR in the initial surgeries were 2.42 (Swine #1) and 3.22 (Swine #2) for the CBD, without the need for surrounding dissection. In the second surgeries, the gallbladders were clearly inflamed without perforation, and the CBDs were visualized with BL-760 with a TBR of 2.83 (Swine #1) and 2.60 (Swine #2).

**Conclusions:**

BL-760 demonstrates high specificity for the biliary tree in an obstructive biliary model. BL-760’s rapid, enhanced visualization has the potential to improve the accuracy of identifying biliary anatomy and enhance cholecystectomy safety.

**Supplementary Information:**

The online version contains supplementary material available at 10.1007/s00464-025-12252-9.

Bile duct injury (BDI) is a dreaded complication of laparoscopic cholecystectomy. Bile duct injuries occur in 0.4–1.5% of laparoscopic cholecystectomies and are a source of patient morbidity and costs to the healthcare system [1]. A major contributing factor to BDI is inadequate visualization of the extra-hepatic biliary ducts, particularly in the case of biliary tree inflammation that is present in many cholecystectomies [1–3]. Biliary tree inflammation is thus associated with an increased risk of BDI [4–8].

At present, there are several options available for surgeons to assist with intraoperative visualization of the biliary tree. Intraoperative cholangiogram (IOC) was first introduced in 1931 by Mirizzi to help visualize choledocholithiasis [9]. Since its introduction, IOC has been widely used for assistance in intraoperative visualization of the biliary tree [10]. However IOC is invasive, technically challenging, increases operative times, and is not associated with lower rates of BDI [11, 12].

Near-infrared (NIR) indocyanine green (ICG) cholangiography has emerged as a commonly used non-invasive tool for assistance in identifying extra-hepatic biliary structures [13–18]. Intravenous (IV) ICG has recently gained popularity and in 2023 was recommended by the European Association of Endoscopic Surgery for use during laparoscopic cholecystectomy [19]. ICG cholangiography during laparoscopic cholecystectomy is associated with higher rates of surgeon satisfaction, decreased operative times, and enhanced visualization of the common bile duct (CBD) [20–22].

ICG cholangiography, however, has several limitations. ICG has a slow onset of action and can only be visualized within the biliary tree 30 min after IV injection. Moreover, ICG must be injected preoperatively [13]. ICG is also limited by its lack of specificity to the biliary tree and by its decreased efficacy in the setting of inflammation [23, 24]. A recent meta-analysis found that ICG does not improve cystic duct (CD) or common hepatic duct (CHD) visualization and does not decrease rates of BDI [22].

Alternative cholangiography options are limited. An alternative use of ICG is by intra-cholecystic (IC) administration [25]. This technique has improved target-to-background ratios (TBRs) for the CD and CBD as compared to IV ICG administration [25]. However, IC ICG leads to increased operative times, can be technically challenging, and provides poor visualization of the CHD [25]. Other technologies such as Methylene Blue and intraoperative ultrasound with saline injection have significant limitations and are rarely used [25, 26].

In light of the limitations of the current cholangiography options, our team previously reported bile-label 760 (BL-760), a novel NIR dye, as an alternative tool for non-invasive intraoperative identification of the biliary tree [27, 28]. BL-760 is a pre-clinical NIR dye that can be visualized with any laparoscope that can visualize ICG. BL-760 is injected intravenously and can be identified in the biliary tree within five minutes with a TBR up to 4.12. After injection, BL-760 can be visualized for over 150 min and is excreted through the gastrointestinal tract. BL-760 is highly specific to the biliary tree and is not visualized in the vasculature.

As part of our previous work, our group observed intraoperative guidance using BL-760 during pre-clinical models of cholecystectomy and hepatectomy [27, 28]. However these studies were not performed in the setting of acute inflammation where, as mentioned, ICG has been shown to be less efficacious at visualizing biliary structures [24].

We thus set out to build on our prior work and create a swine model of biliary inflammation. We attempted to induce cholangitis and cholecystitis, pathologies that begin with biliary obstruction, leading to biliary stasis and subsequent inflammation [29]. Our approach was consistent with previously described swine models in which biliary inflammation was created by either clipping or suture ligating the CBD to induce biliary obstruction [30, 31].

In summary, this study aimed to assess the intraoperative detection of the extra-hepatic biliary tree with BL-760 in a swine model of induced biliary obstruction and inflammation.

## Materials and methods

BL-760 is a heptamethine cyanine NIR dye in the early phases of the regulatory pathway, with a goal of starting clinical trials in the next year. Following IV injection BL-760 can be visualized within the biliary tree in the near-infrared wavelengths. The synthesis and characterization of BL-760 is described in our group’s previous work [27, 32, 33].

For this study, BL-760 was produced by a third-party vendor (Lot no. AL1145-9-C, Alchem Laboratories Corporation, Alachua, FL) stored at room temperature, and reconstituted with saline immediately prior to injection. We injected 5 µg/kg of BL-760, at a concentration of 0.5 mg/ml. For laparoscopic imaging, we used a 10 mm 30-degree NIR-capable laparoscope with a working distance of approximately 3–5 cm (HOPKINS RUBINA 30° NIR/ICG, Karl Storz Endoscopy), connected to an FDA-cleared dual white/NIR light source (OPTOVISION Endoscopic Light Source Unit, Optosurgical, LLC).

To investigate the potential use of BL-760 in the setting of biliary inflammation, we performed a survival swine study in two 30 kg female Yorkshire swine. Swine were chosen because of the similarities between swine and human biliary anatomy and because swine were used in previous NIR biliary tree pre-clinical studies [34–36]. All animal procedures were approved by our Institutional Animal Care and Use Committee (IACUC #30591).

After arrival at the animal facility the swine were acclimated for 24 h and individually housed. The swine were fasted for 12 h before surgery and were administered preoperative Cefazolin. Anesthesia was induced with Ketamine and Xylazine and inhaled Isoflurane was used to maintain anesthesia. The swine were intubated, positioned supine on the operating table, and their abdomens were prepared with Betadine. Heart rate, cardiac rhythm, oxygen saturation, and rectal temperature were continuously monitored during the surgeries. All sterile precautions were performed.

Each swine underwent two surgeries. In each initial surgery, laparoscopic access was obtained and a 12 mm infraumbilical port was placed under direct visualization. Three additional ports were placed: a 12 mm port in the epigastrium, a 5 mm port in the right lateral subcostal position, and a 5 mm port in the right subcostal mid-clavicular line. After laparoscopic access was obtained, BL-760 was injected intravenously. Laparoscopic clipping of an extra-hepatic biliary duct was performed (the CD in Swine #1 and the CBD in Swine #2) using an endoscopic clip applier (LIGACLIP™ Endoscopic Rotating Multiple Clip Applier, ETHICON). Clipping was performed after minimal surrounding dissection. Port sites were closed with absorbable sutures and sterile dressings were placed. The swine were awoken from anesthesia, administered a one-time dose of Buprenorphine for pain control, and continuously monitored until ambulatory.

Postoperatively, the swine were administered Cefazolin and Carprofen every 12 hours. Vital signs were checked twice daily. On postoperative day three each swine returned to the operating room for the second surgery. Laparoscopic access was obtained through the previously placed incisions. BL-760 was then injected intravenously, and a laparoscopic cholecystectomy was performed. Following the second surgeries, the swine were euthanized under general anesthesia per our IACUC protocol. To minimize the number of swine utilized in our study no control group was used.

To assess the fluorescence of BL-760 TBR was calculated. The following formula was used; TBR = (target fluorescence intensity-noise)/(liver fluorescence intensity-noise). Image analyses and calculations were performed using ImageJ [37].

## Results

### Swine #1

**Fig. 1:**
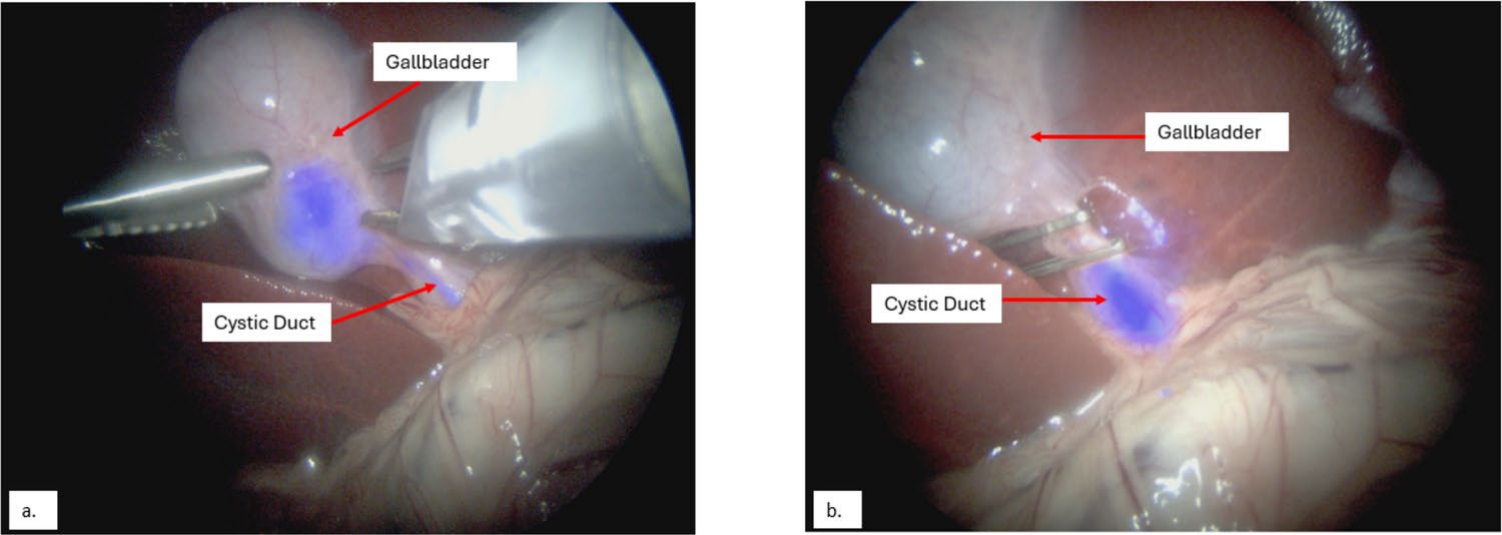
Swine #1—Images of the gallbladder neck and cystic duct during the first surgery before (**a**) and after (**b**) cystic duct clipping with BL-760 overlay

**Fig. 2:**
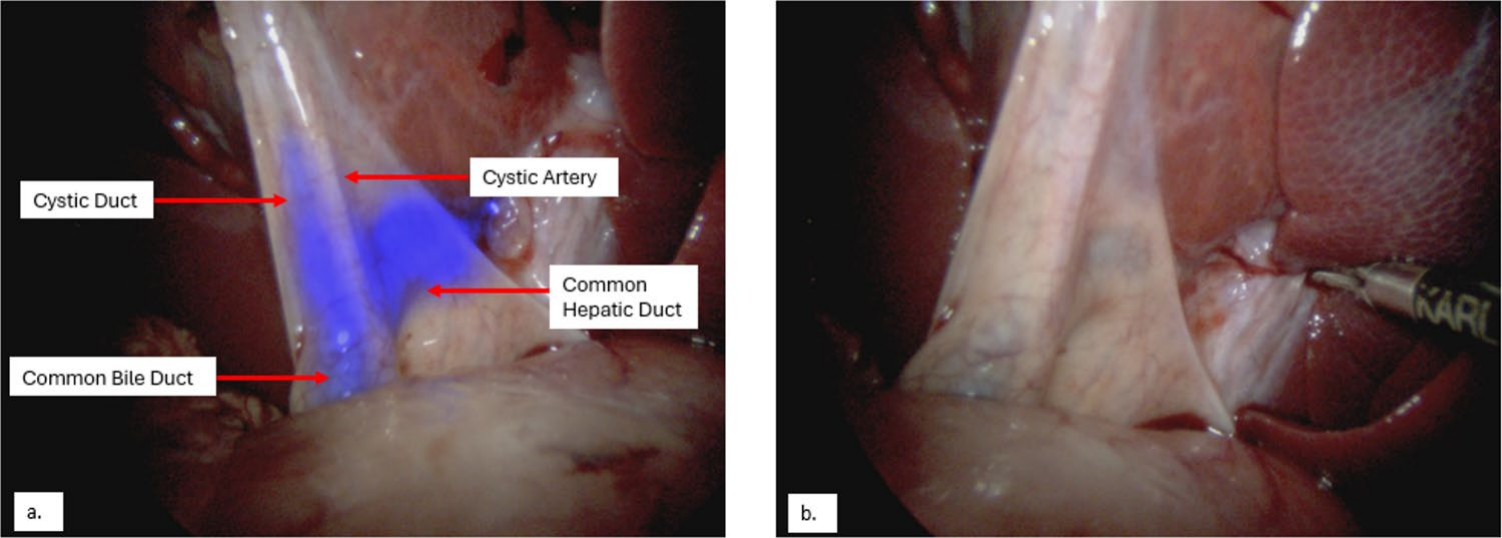
Swine #1—Images of the extra-hepatic biliary tree during the second surgery with (**a**) and without (**b**) BL-760 overlay

In the initial surgery, laparoscopic BL-760 guided cystic duct and artery clipping was performed without complication. The CD was visualized with a TBR of 2.50 (Fig. 1) and the CBD was visualized with a TBR of 2.42. Postoperatively, the swine was afebrile, non-tachycardic, had a normal oxygen saturation, and tolerated a regular diet. In the second surgery, on laparoscopy, there was no evidence of peritonitis or peritoneal adhesions and the gallbladder was clearly inflamed without perforation. With BL-760, the CD was visualized with a TBR of 2.83 and the CBD was visualized with a TBR of 3.88 without the need for surrounding dissection (Fig. 2). Because the CD was clipped no BL-760 entered the gallbladder. An uncomplicated cholecystectomy was performed aided by BL-760 fluorescence of the biliary tree.

### Swine #2

**Fig. 3:**
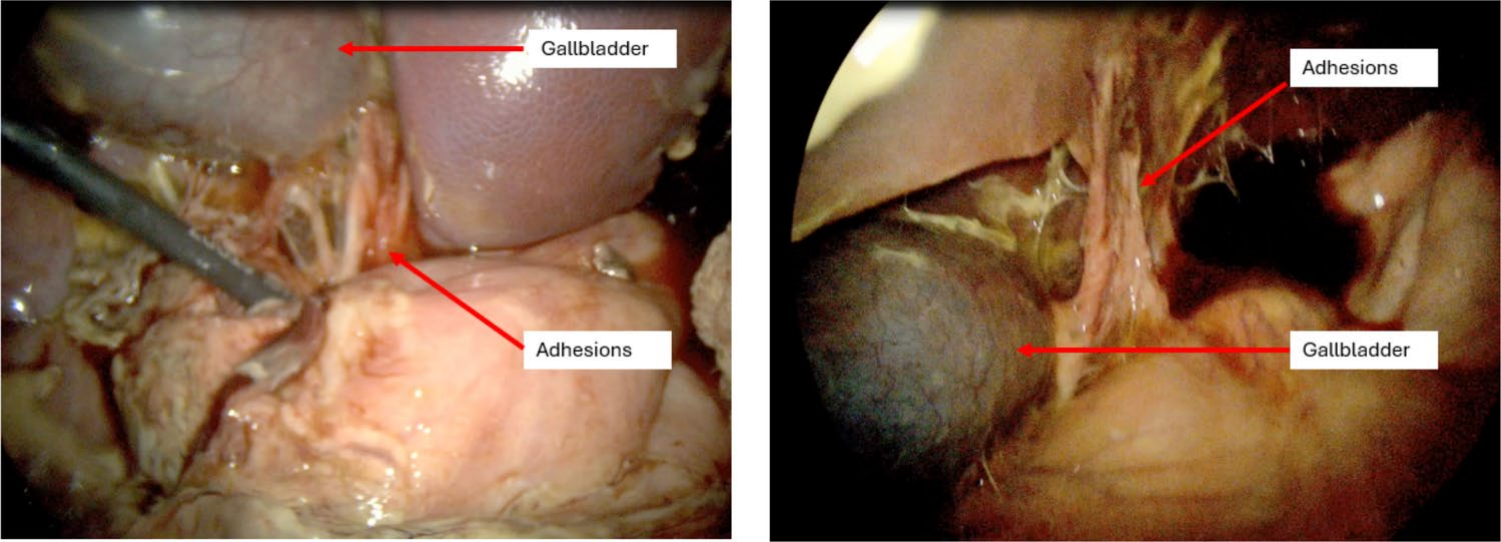
Swine #2 – Initial laparoscopic imaging of the abdomen during the second surgery, revealing significant intraperitoneal adhesions and inflammation

**Fig. 4:**
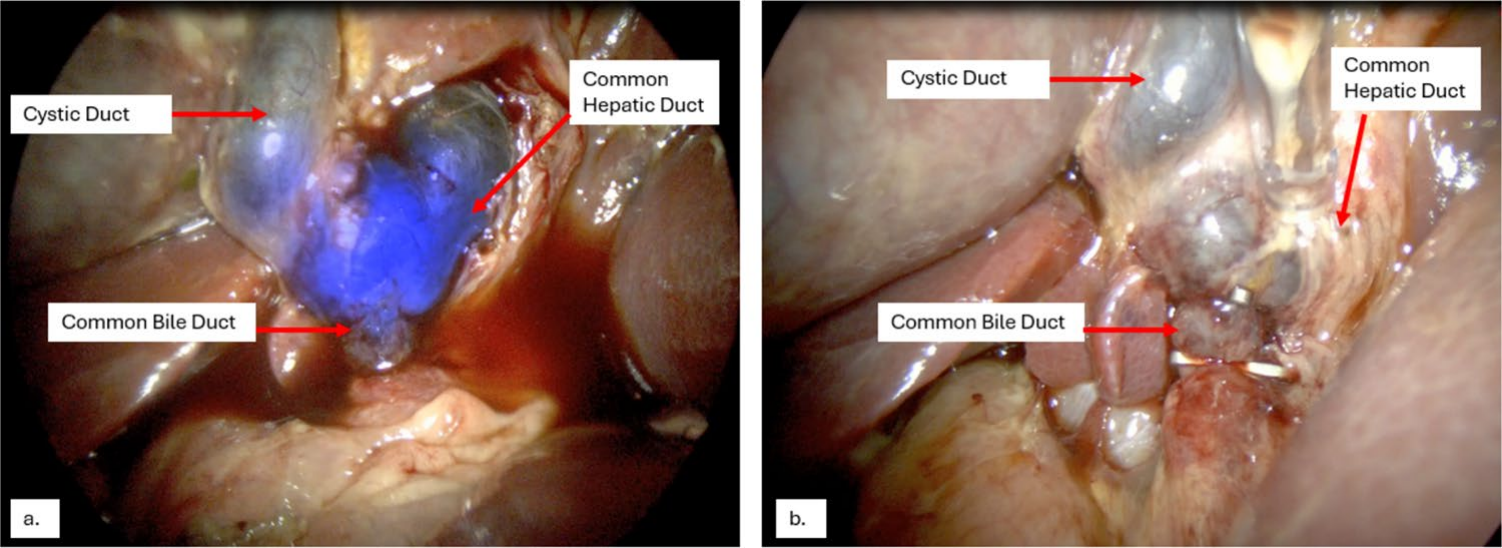
Swine #2—Images of the extra-hepatic biliary tree during the second surgery with (**a**) and without (**b**) BL-760 overlay

In the initial surgery, laparoscopic BL-760 guided CBD clipping was performed without complication. The CD was visualized with a TBR of 3.82 and the CBD was visualized with a TBR of 3.22 (Table 1). Postoperatively, the swine was afebrile, non-tachycardic, had a normal oxygen saturation, but had minimal oral intake. In the second surgery, on laparoscopy there were significant intraperitoneal adhesions and inflammation and the extra-hepatic biliary tree, including the gallbladder, was dilated and inflamed up to the previously placed clips (Figs. 3, 4). With BL-760, the CD was visualized with a TBR of 3.75 and the CBD was visualized with a TBR of 2.60. An uncomplicated cholecystectomy was performed aided by BL-760 fluorescence of the biliary tree.

**Table 1:** Target-to-background ratios for the extra-hepatic biliary ducts during each surgical procedure

	Common Bile Duct	Cystic Duct	Common Hepatic Duct
Swine #1 Surgery #1	2.42	2.50	2.45
Swine #1 Surgery #2	2.83	3.88	2.83
Swine #2 Surgery #1	3.22	3.82	2.28
Swine #2 Surgery #2	2.60	3.75	2.07

Both swine tolerated the procedures well, without intraoperative complications. There were no vital sign changes after BL-760 injection. In all four surgeries BL-760 was visible within five minutes of injection and throughout the procedure. The half-life of BL-760 is not yet determined, but in each second surgery no BL-760 from the prior surgery was visualized.

## Discussion

This study demonstrates that BL-760 may be a viable, highly specific tool for extra-hepatic biliary duct visualization in the setting of biliary obstruction and inflammation. Our results correlate with our prior work that showed BL-760 as a viable tool for bile duct identification during swine hepatectomy and cholecystectomy [27, 28].

BL-760 may present several advantages over the currently available tools for assistance with identifying the extra-hepatic biliary tree. While IOC is a valuable tool during laparoscopic cholecystectomy, it is invasive, necessitating access to the biliary tree and flushing the tree with radiolucent dye. This exposes the biliary system to the introduction of foreign material. Additionally, IOC exposes the patient and staff to ionizing radiation, increases operative time, and requires specialized training [5, 12]. BL-760, similar to other NIR dyes, allows for quick, non-invasive, and radiation-free intraoperative visualization of the extra-hepatic biliary tree. Ideally, biliary-specific NIR dyes can be used to help determine when IOC and transcystic CBD exploration are indicated [38].

BL-760 also may present several advantages over ICG, the most commonly used NIR dye for assistance with intraoperative visualization of the biliary tree. BL-760 is specific to the biliary tree. In contrast, ICG is first visualized in the vascular system, then the liver, and lastly in the biliary tree [39]. This sequence limits ICG’s biliary specificity as the intense fluorescence of the liver, blood vessels, and even intraoperative bleeding can obscure biliary tree identification [25]. ICG’s lack of biliary specificity can also affect its ability to recognize bile leaks. Our group’s prior pre-clinical work showed BL-760 as superior to ICG at recognizing bile leaks [28].

BL-760 can be visualized within five minutes of injection. In contrast, ICG has a slow onset of action and can only be visualized within the biliary tree 30 min after IV injection. A recent international Delphi study recommends ICG injection at least 45 min before the start of laparoscopic cholecystectomy [40]. This slow onset can limit ICG’s efficacy, for example when an unexpected difficult dissection arises intraoperatively. ICG’s optimal visualization time is also variable, with studies citing optimal visualization ranging from 3 to 15 h after injection [13, 41].

BL-760 has consistent visualization and TBR both in the presence and in the absence of inflammation. In this study the average TBRs for the extra-hepatic biliary tree were similar for both the first surgeries (2.79), performed in the absence of inflammation, and the second surgeries (3.00), performed in the presence of inflammation. These TBR values correlate with our previous study evaluating BL-760 during laparoscopic cholecystectomy, albeit in the absence of inflammation [27]. In contrast, ICG’s efficacy is limited in the setting of biliary tree inflammation, a significant consideration in light of biliary tree inflammation’s association with higher rates of BDI [24, 40].

Other technologies such as IC ICG, Methylene Blue, and intraoperative ultrasound with saline injection have emerged as potential alternatives for intraoperative biliary ductal visualization [25, 26]. However, these alternatives each have significant limitations and are rarely used. Accordingly, a less invasive tool such as BL-760, which potentially allows for specific visualization of the biliary tree in the setting of inflammation, can be extremely valuable.

NIR dyes will have a significant role in the future of artificial intelligence (AI) guided surgery [42]. Yin et al. recently used ICG to help implement a deep learning system to identify extra-hepatic biliary structures [43]. Ryu et al. simultaneously used AI and an NIR fluorescent urethral catheter for intraoperative ureter identification during colorectal surgery [44]. BL-760’s specificity for the biliary tree can potentially serve as a tool to help train future deep learning and AI models.

Our swine CBD and CD clipping models have several attributes that make them potentially useful for future education and research. Our models induced an obstructed and inflamed biliary tree without systemic infection: our CD clipping model caused localized gallbladder inflammation, while our CBD clipping model caused extra-hepatic biliary dilation and a widespread intraperitoneal inflammatory response. These models can be utilized for future laparoscopic cholecystectomy training for surgical trainees, where there is a skill and knowledge gap [45, 46]. They can also be used for continued research assessing NIR dye efficacy in the setting of biliary tree inflammation.

Our study had multiple limitations. There was only one swine in each group, therefore, our findings are preliminary and represent proof-of-concept observations. We also did not use ICG concomitantly, which could have served as a positive control; we did not want to obscure BL-760 visualization by injecting an additional dye, and we also did not want to extend operative time. We did not use a control group. Despite the similarities between swine and human biliary anatomy, our study still has the generalizability limitations of an animal model. Additionally, although our model induced biliary obstruction, much of the inflammation encountered in human cholecystitis is associated with adipose tissue, which we likely did not capture. Further validation in larger cohorts and more clinically representative models is needed to confirm BL-760’s efficacy in the setting of inflammation.

BL-760 is in the early phase of the regulatory pathway. Our initial studies used 90–100 µg/kg to ensure robust biliary fluorescence and establish proof-of-concept [27, 28]. However, subsequent pharmacokinetic and imaging studies demonstrated that BL-760 achieves a strong biliary-specific signal at significantly lower doses. For translational development, we adopted 5 µg/kg to minimize systemic exposure and align with FDA expectations for the lowest effective dose. This strategy maximizes safety margin, facilitates Good Laboratory Practice toxicology interpretation, and ensures an appropriate bridge to human dose extrapolation.

Before transitioning to clinical trials, more work is needed to better understand BL-760’s clinical usability, versatility, and safety. We need to better understand BL-760’s optimal dosing and optical setup, including lens configuration and excitation intensity. Additionally, we need to fully characterize the tissue penetration of BL-760 in relation to currently available dyes. Based on BL-760’s physiochemical properties and its emission in the NIR spectrum, we anticipate a penetration depth comparable to, or potentially greater than, that of ICG, given its relatively high quantum yield. However, this needs to be quantified and validated. We will also explore the potential of IC BL-760 administration and BL-760’s potential to detect bile duct stones. Lastly, BL-760’s safety profile needs to be fully understood. Early bile duct identification is essential to preventing BDIs, and BL-760 may prove to be a useful tool with significant clinical benefit.

BL-760 demonstrates high specificity for the biliary tree in an obstructive biliary model. BL-760’s rapid onset and enhanced visualization has the potential to improve the accuracy of biliary anatomy delineation and increase cholecystectomy safety.

## Supplementary Information

Below is the link to the electronic supplementary material.

Supplementary file1 (MOV 313775 KB)
